# Socio-economic behavioural indicators of falciparum malaria parasitaemia and moderate to severe anaemia among pregnant women attending antenatal clinics in Lagos, Southwest Nigeria

**DOI:** 10.1186/s12936-020-03462-8

**Published:** 2020-11-07

**Authors:** Adeola Y. Olukosi, Abiodun Olakiigbe, Olusola Ajibaye, Bassey A. Orok, Olugbenga O. Aina, Samuel K. Akindele, Olajumoke O. Akinyele, Adebayo T. Onajole, Samson T. Awolola, Tolulope Arowolo, Bamigboye M. Afolabi

**Affiliations:** 1 Nigerian Institute of Medical Research, 6, Edmund Crescent, PMB 2013, Yaba, Lagos, Nigeria; 2 College of Medicine, University of Lagos, Idi-araba, Lagos, Nigeria; 3 Health, Environment and Development Foundation, Surulere, Lagos, Nigeria

**Keywords:** Malaria, Anaemia, Pregnancy, Behavioural factors, Southwest Nigeria

## Abstract

**Background:**

Incidence of malaria and anaemia are of public health importance especially in pregnant women in endemic regions, due to the negative health consequences to the mother and fetus. This study aimed to assess the pattern of falciparum malaria infection and anaemia, based on malaria prevention methods practiced by participants.

**Methods:**

A semi-structured tool was used to capture information on demographic, socio-economic and malaria prevention practices from 113 pregnant women attending antenatal clinics in 2 peri-urban health facilities in Lagos, southwest Nigeria. Malaria microscopy was conducted and haematocrit was measured. Logistic regression analysis was performed on the data collated from the survey.

**Results:**

The prevalence of anaemia among pregnant women was 87.2%. The mean (± sd) packed cell volume (PCV) (%) of the 22 (19.5%) infected subjects (26.8 ± 6.6), was significantly lower (t = −2.60, *P* value = 0.007) than that of the 91 (80.5%) uninfected subjects (30.8 ± 6.0). The prevalence of infection was highest in the 3rd trimester (n = 40, 35.4%) at 27.5% (11/40) and among those in their first pregnancy (n = 32, 28.3%) at 25.0% (8/32). There was a significant difference (t = −2.23, P-value = 0.01) in the mean PCV % of pregnant women who consumed herbal teas in pregnancy (28.2 ± 5.2) compared to those who did not (30.8 ± 6.6). Regression analysis showed that first pregnancy, anti-malarial use and insecticide-treated nets use the night before study had increased odds of malaria infection in participants (OR = 1.35, P = 0.006, 95% CI 0.52−2.49; OR = 2.3, P = 0.005, 95% CI 0.14−0.41; OR = 1.92, P = 0.001, 95% CI 0.62−5.98) while intermittent preventive treatment (IPT) participation and formal education were strongly and significantly associated with lower risk of parasitaemia (OR = 0.95, P = 0.025, 95% CI 0.41−2.26; OR = 0.44, P = 0.005, 95% CI 0.34−10.50).

**Conclusion:**

Interventions that will reduce malaria and moderate to severe anaemia, especially in a first pregnancy, should include education on the correct use of long-lasting insecticide-treated bed nets (LLIN), IPT and the dangers of herbal teas in pregnancy.

## Background

Pregnant women are one of the vulnerable groups especially susceptible to malaria because of the sub-optimal immune status conferred by increased steroid levels in pregnancy [1]. Mosquitoes are more attracted to pregnant women and parasitized erythrocytes are sequestered in the placenta by adhesion to the chondroitin sulfate A expressed in the placenta [2] of pregnant women. In areas of stable transmission, it is estimated that malaria during pregnancy causes up to 10,000 maternal deaths each year, mainly as a result of severe anaemia, and accounts for approximately 8–14% of low birth weight (LBW), and 3–8% of infant mortality [3]. Infectious diseases, including malaria, iron and other micronutrient deficiencies, are contributing factors to this pervasive global situation of anaemia, the commonest medical condition in pregnancy which has a global prevalence of 41.8% and rises to as high as 75% in The Gambia [4]. Anaemia prevalence in Nigeria is 58% from recent reports, making it a continuing severe situation [4, 5]. Anaemia is the major contributory or sole cause in 20–40% of maternal deaths [6]. Mechanisms of anaemia causing malaria include lysis of infected and uninfected red blood cells (RBCs), splenic sequestration of RBCs, dyserythropoietic, and bone marrow suppression [7].

Control measures target to be protective of this section of the population in endemic countries to avoid many of the associated complications to the outcome of pregnancy [8]. The use of long-lasting insecticide-treated nets (LLINs), intermittent preventive treatment (IPT) with sulfadoxine-pyrimethamine (SP) and prompt treatment of confirmed malaria are the core strategies for control of malaria in pregnancy in Nigeria [9]. These interventions are delivered in health facilities with an emphasis on the promotion of focused antenatal care [10, 11].

The percentage of women that attend antenatal care (ANC) in health facilities is generally low at 58% across the country, with the lowest rates of ANC attendance observed in the northern states. All pregnant women are to receive at least 3 doses of SP treatments during their ANC visits under directly observed therapy (DOT) and its implementation appears to be fairly well integrated at primary health facility level across the country. LLIN utilization for pregnant women improved from 5% in 2008 to 10% in 2010 and 49% in 2015 [12]. Similarly, the percentage of pregnant women that received at least 2 doses of IPT improved from 6.5% in 2008 [13] to 13.2% in 2010 [14] and 37% in 2015 [15]. Rational use of anti-malarials to treat malaria in pregnancy is well outlined in the Federal Ministry of Health’s National Guidelines for diagnosis and treatment of malaria but deterrents of access to treatment make it difficult to put guidelines into use. Several other practices and factors have been implicated to contribute to malaria infection and anaemia status, including state of education, use of unrecommended herbal medication and the physiological status of the individual concerned. The prevailing factors and practices that predispose to malaria and anaemia in pregnancy are the subject of this study. This study has assessed the prevalence of malaria parasites, moderate to severe anaemia, and factors associated with their risk to pregnant women attending antenatal clinics in two General Hospitals in Ikorodu local government area of Lagos State in Nigeria, to guide intervention with malaria control measures.

## Methods

A detailed description of the study population, sample size determination and ethical considerations have been published in an earlier paper [16]. Briefly, a cross-sectional survey was undertaken amongst the study population comprising pregnant women attending antenatal clinic at Ijede General Hospital, Ijede (N06° 34.076 E003° 35.637′) and Ikorodu General Hospital both in Ikorodu Local Government Area **(**LGA), Lagos State in the period January to April 2009. The inclusion criteria were women with fever or history of fever in 24–48 h preceding presentation at the hospital and those that signed or thumb-printed the consent form. Patients with signs of severe malaria were excluded from the study.

### Ethical issues

Ethical approval was obtained from the Nigerian Institute of Medical Research Institutional Review Board. All work was performed according to the guidelines for human experimentation in clinical research and the Helsinki declaration.

### Data and sample collection

Structured questionnaires were administered to each pregnant woman designed to collect demographic data, history of drug use, participation in IPT, LLIN possession, information on gestational age, history of obstetrics, and history of febrile illness. Venous (0.5 ml) blood sample was collected from a peripheral vein in each participant by a trained laboratory scientist or technician into a microtainer labelled EDTA bottles. Haematocrit tubes were three-quarters filled and spun at 12,000—15,000×*g* for 5 min on a haematocrit centrifuge (Hawksley, England). Packed cell volume (PCV) percentage was read of the Haematocrit Reader (Hawksley, England).

To prepare thick and thin blood films, 3 drops of blood were spread in a circle of about 1-cm diameter at one end of the slide, and about 1 cm from that, one drop of blood for the thin film was spotted and spread in a thin film. The films were allowed to dry for about 15 min before fixing the thin film with methanol. The slides were allowed to air dry until the next day. Slides were then stained with 3% Giemsa for 45 min, rinsed, dried, and examined under the microscope as detailed in a previous publication [17]. The results are reported as the number of parasites per high-powered field (HPF) or the number of parasites per 200 HPF. The parasite density per µl is calculated by multiplying the number per HPF by 500, based on assumptions that 5–8 µl of blood is used in making a thick blood film and that 0.002 µl of blood is in an HPF, i.e. 10 X eyepiece, 100 X objective [18].

Anaemia status of the participants was defined using the World Health Organization (WHO) haematocrit cut-off for mild, moderate and severe anaemia based on age and gender. The demographic information of the participants was also recorded.

### Data analysis

Inferential and descriptive statistics were used for data presentation. Data entered into Microsoft Excel 2010 were imported into SPSS 20.0 for Windows for analysis. Variables considered in the analysis were related to the presence and densities of malaria parasites, fever, anaemia, and participants’ demographics. Anaemia was classified as mild (PCV 24.0– < 32.0%) or moderate (PCV 18.0- < 24.0%) or severe (PCV < 18.0%). Proportions were compared by calculating Chi square, Fisher’s exact or Mantel–Haenszel tests as appropriate. Normally distributed continuous data were compared by t-test and analysis of variance. Data not conforming to a normal distribution were compared by the Mann–Whitney U tests and the Kruskal–Wallis tests (or by Wilcoxon ranked sum test). P values less than 0.05 were considered statistically significant. Unadjusted and adjusted odds ratios with 95% confidence intervals (CIs) for the associations between exposures and moderate to severe anaemia were computed using logistic regression methods.

## Results

### Participants’ profile and preventive behavioural characteristics of the study population

Detailed descriptions of the socio-clinical profiles of 113 pregnant women, whose mean (± sd) age was 30.0 years (4.7) are as described in Table 1. Of these 113 pregnant women, 4 (3.6%) were aged < 20 years. Only 5 (4.4%) had no formal education whereas 51 (45.1%) had a minimum of secondary education. Fifty-four (47.8%) were traders, 17 (15.0%) were students and 11 (9.7%) were housewives. In all, 32 (28.3%), 32 (28.3%) and 49 (34.4) were primigravida, secundigravida and multigravida, respectively, while 19 (16.8%), 54 (47.8%) and 40 (35.4%) were in their first, second and third trimester, respectively. Those that had moderate to severe anaemia were 80 (70.8%) contrasting with 33 (29.2%) that presented with no or mild anaemia. Of all the study subjects, only 22 (19.5%) were infected with falciparum malaria parasites among whom 9 (40.9%) were aged 26–30 years, 12 (54.5%) with secondary education, 12 (54.5%) who were traders, 11 (50.0%) in their third trimester, and 10 (45.4%) who were multigravida. A total of 19 (86.4%) of those infected with falciparum malaria parasites (FMP) also had moderate to severe anaemia. Those aged 26-30 years were 1.64 more likely to be infected (χ^2^ = 1.02, P-value = 0.31, OR = 1.64, 95% CI 0.63, 4.28) than all other ages and pregnant women without formal education were 2.9 times more likely to be infected than others with education (χ^2^ = 0.37, P-value = 0.54, OR = 2.90, 95% CI 0.46, 18.73). Those in their third pregnancy were 1.60 times more likely to be infected with FMP compared to women of other gravidities (χ^2^ = 0.86, P-value = 0.35, OR = 1.60, 95% CI 0.60, 4.28) while those in the first trimester were 2.14 times likely to be infected with FMP than those in other trimesters (χ^2^ = 2.52, P-value = 0.11, OR = 2.14, 95% CI 0.83, 5.50) (Table 1). Those with moderate to severe malaria were 3.1 times likely to be infected than those with mild or no anaemia (χ^2^ = 2.33, P-value = 0.13, OR = 3.1, 95% CI 0.85, 11.36) (Table 1).

**Table 1:** Distribution of social and clinical profile of infected and non-infected pregnant women participants attending Ikordu and Ijede General Hospital, Lagos State

Variable	Item	Freq. (%)	Mean (±sd) of age	Infected		Not infected		χ²	P-value	OR	95% CI
				Freq.	%	Freq.	%				
Age	All	113 (100.0)	30.0 (4.7)	22	19.5	91	80.5	-	-	-	-
	≤ 20	4 (3.6)	18.5 (3.0)	1	25.0	3	3.3	0.00	1.00	1.40	0.14, 14.11
	21.25	24 (21.2)	23.8 (1.2)	6	25.0	18	19.8	0.59	0.44	1.52	0.52, 4.44
	26–30	36 (31.9)	28.1 (1.4)	9	25.0	27	29.7	1.02	0.31	1.64	0.63, 4.28
	31–35	31 (27.4)	33.0 (1.4)	4	12.9	27	29.7	0.67	0.41	0.53	0.16, 1.70
	> 35	18 (15.9)	39.3 (3.0)	2	11.1	16	17.6	0.42	0.51	0.47	0.10, 2.21
Education	No formal	5 (4.4)	36.6 (6.9)	2	4.0	3	3.3	0.37	0.54	2.9	0.46, 18.73
	Primary	13 (11.5)	33.5 (6.9)	0	0.0	13	14.3	2.29	0.13	0.0	Undefined
	Secondary	51 (45.1)	28.7 (59)	12	23.5	39	42.9	0.97	0.32	1.6	0.63, 4.08
	Tertiary	44 (38.9)	29.6 (4.3)	8	18.2	36	39.6	0.08	0.78	0.87	0.33, 2.29
Occupation	House wives	11 (9.7)	25.8 (3.5)	1	9.1	10	11.0	0.26	0.61	0.39	0.05, 3.18
	Labor work	6 (5.3)	30.7 (6.4)	1	18.5	5	5.5	0.00	1.00	0.82	0.09, 7.39
	Office work	25 (22.1)	32.3 (5.1)	3	12.0	22	24.2	0.61	0.43	0.50	0.13, 1.83
	Students	17 (15.0)	25.3 (4.0)	5	29.4	12	13.2	1.25	0.26	1.93	0.60, 6.22
	Traders	54 (47.8)	31.1 (5.8)	12	22.2	42	46.1	0.50	0.48	1.40	0.55, 3.57
Trimester	First	19 (16.8)	28.6 (5.9)	3	15.8	16	17.6	0.02	0.90	0.74	0.19, 2.80
	Second	54 (47.8)	30.2 (5.6)	8	14.8	46	50.5	1.42	0.23	0.56	0.21, 1.46
	Third	40 (35.4)	30.3 (6.1)	11	27.5	29	31.9	2.52	0.11	2.14	0.83, 5.50
Gravidity	Primigravida	32 (28.3)	26.8 (5.2)	8	25.0	24	26.5	0.86	0.35	1.60	0.60, 4.28
	Secundigravida	32 (28.3)	27.3 (3.7)	4	12.5	28	30.8	0.83	0.36	0.50	0.15, 1.61
	Multigravida	49 (43.4)	33.7 (5.1)	10	20.4	39	42.9	0.05	0.83	1.11	0.44, 2.83
Anemia	None/Mild	33 (29.2)	30.3 (7.2)	3	9.0	30	33.0	2.33*	0.13	3.11	0.85, 11.36
	Moderate/ Severe	80 (70.8)	29.8 (5.2)	19	23.8	61	67.0				

**Fig. 1:**
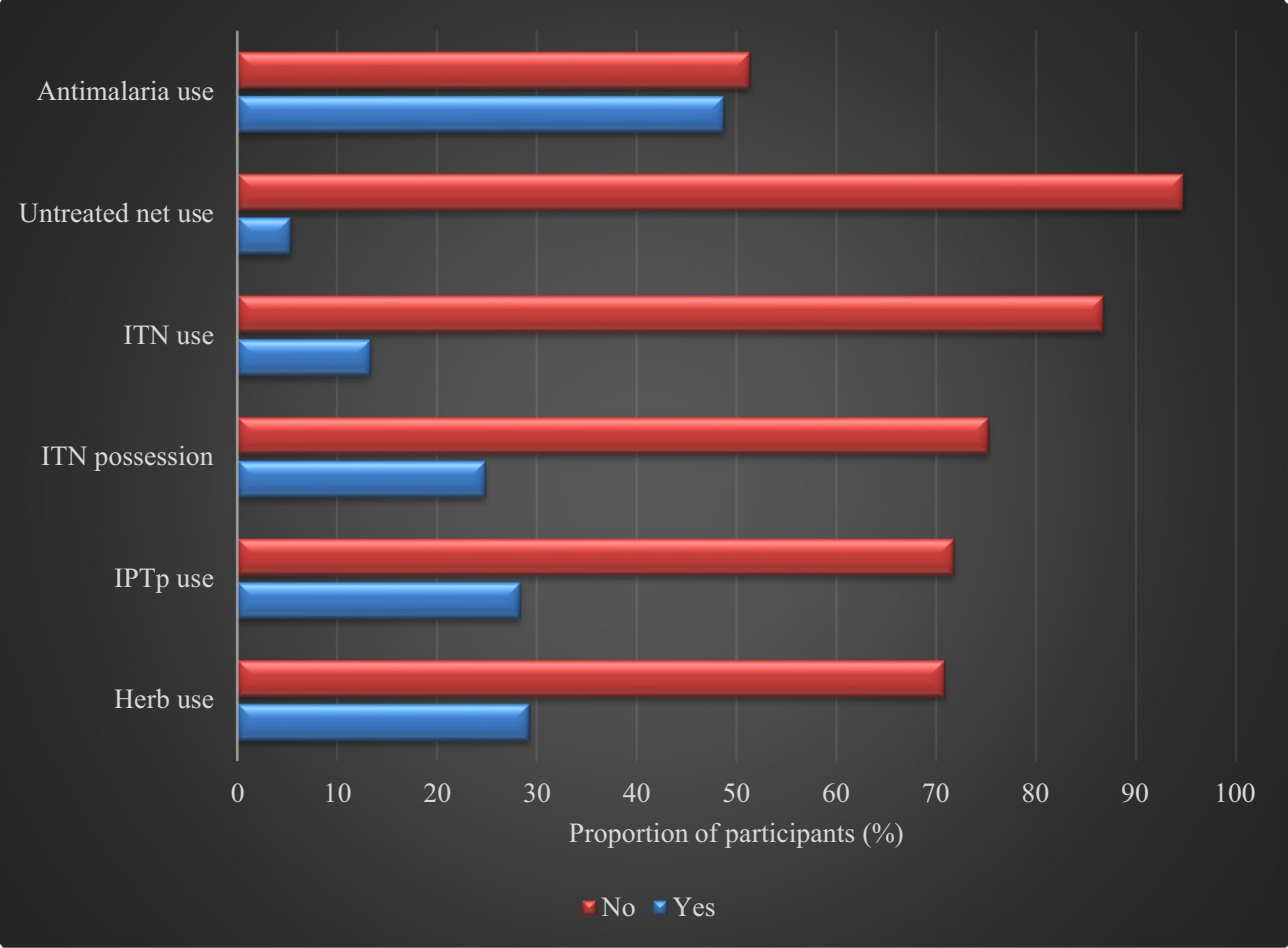
Treatment and prevention measures practiced amongst pregnant women participants attending Ikordu and Ijede General Hospitals in Lagos, Nigeria

The frequency of treatment and preventive measures practiced by participants as displayed in Fig. 1 shows that drug treatment was the most commonly practiced malaria control measure. More than 55% of participants had used anti-malarial drugs of one type or another including, artemisinin combination therapy (ACT) (51%), chloroquine or SP (38%) and other anti-malarial medications (38%).

Association of prevention method, parity and age group presence or non-presence, with peripheral parasitaemia during pregnancy in a univariate and multivariate analysis is presented in Table 2. In the univariate analysis, women in their first pregnancy were 1.35 times more likely to have parasites compared to those in their second or more pregnancy (P-value = 0.006, 95% CI 0.52–2.49). Women under age 20 years were 1.30 times more likely to have parasites compared to women over 20 years old (P-value = 0.05, 95% CI 0.14–12.20). LLIN use was 1.92 times more likely to confer parasitaemia than non-use (P = 0.001, 95% CI 0.62–5.98) and anti-malaria use in pregnancy, 2.3 times more likely to have malaria parasite than women that did not take anti-malaria (P-value, 0.005, 95% CI 0.14–0.41). On the other hand, IPT participation, formal education and herb use conferred a reduced risk of malaria infection compared to non-IPT participation, non-educated, and no herb use, respectively. The odds ratio values in the multivariate analysis follow the same trend as that in the univariate analysis (Table 2).

**Table 2:** Factors associated with peripheral parasitemia during malaria in pregnancy using univariate and multivariate analysis

Variable	Peripheral parasitaemia % (Positive/total)	Univariate		Multivariate	
		Adjusted OR (95% CI)	P-value	Adjusted OR (95% CI)	P-value
1st pregnancy	25.0 (8/32)	1.35 (0.52–2.49)	0.006	1.22 (0.49–2.12)	0.008
2nd or further pregnancies	18.5 (15/81)	1		1	
Age < 20 years	25.0 (1/4)	1.30 (0.14–12.20)	0.052	1.24 (0.05–10.91)	0.092
Age ≥ 20 years	19.3 (21/109)	1		1	
LLIN use	33.3 (5/15)	1.92 (0.62–5.98)	0.001	1.91 (0.56–587)	0.007
No LLIN use	17.3 (17/98)	1		1	
Anti-malarial use	27.3 (15/55)	2.3 (0.14–0.41)	0.005	0.37 (0.24–0.55)	0.031
No anti-malarial use	12.0 (7/58)	1		1	
IPTp participation	18.8 (6/32)	0.95 (0.34–2.64)	0.060	0.85 (0.41–2.26)	0.025
No IPTp participation	19.8 (16/81)	1		1	
Herb use	18.2 (6/33)	0.91 (0.33–2.53)	0.026	0.94 (0.40–2.24)	0.231
No herb use	20 (16/80)	1		1	
Formal education	17.6 (19/108)	0.44 (0.34–10.50)	0.005	0.64 (0.48–5.66)	0.002
No formal education	40.0 (2/5)	1		1	

**Fig. 2:**
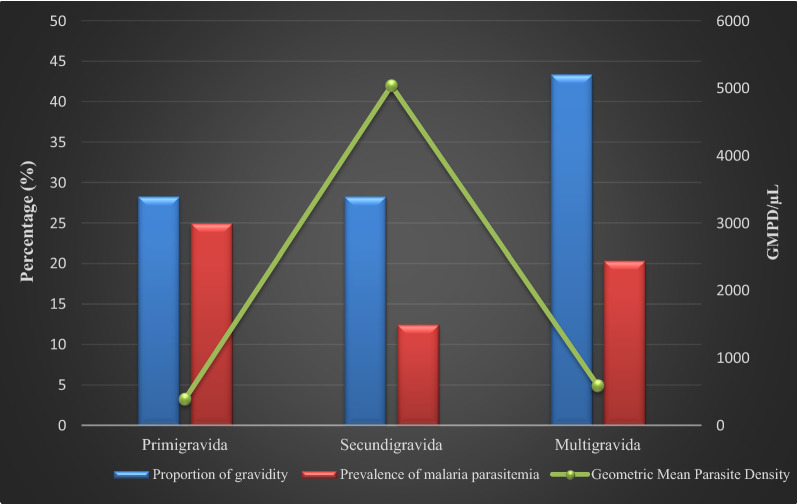
Malaria prevalence and parasitaemia (GMPD) according to the gravidity of pregnant women participants attending Ikordu and Ijede General Hospitals in Lagos, Nigeria

**Fig. 3:**
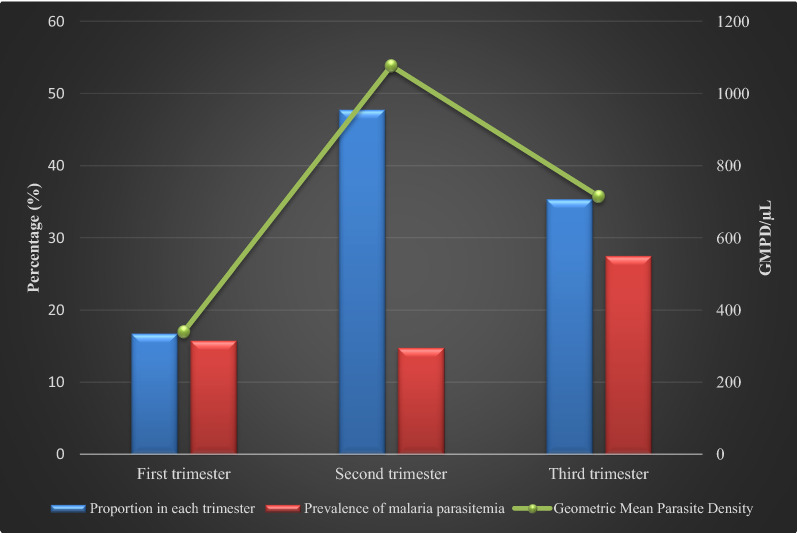
Malaria prevalence and geometric mean parasite density (GMPD) according to gestational ages of pregnant participants attending Ikordu and Ijede General Hospitals, in Lagos, Nigeria

**Fig. 4:**
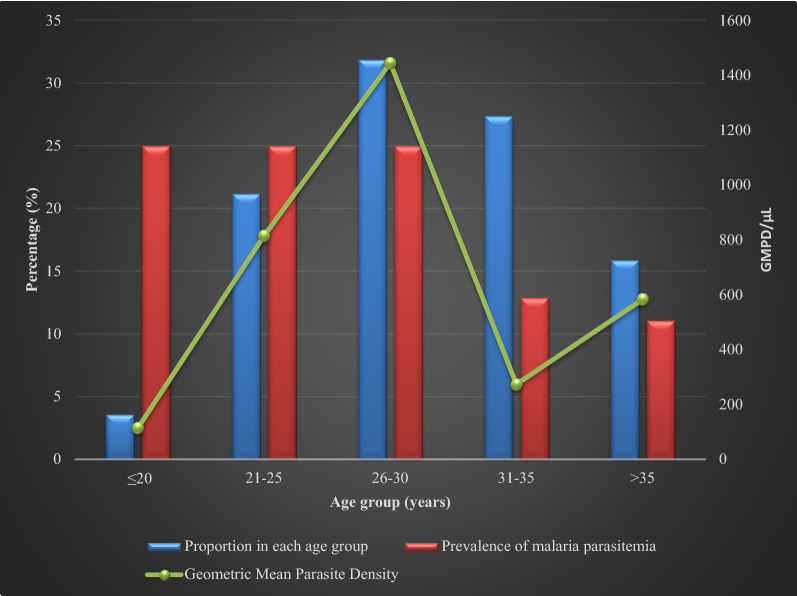
Malaria prevalence and geometric mean parasite density (GMPD) according to age groups of pregnant women participants attending Ikordu and Ijede General Hospitals, in Lagos, Nigeria

Figures 2, 3 and 4 are a graphical representation of malaria prevalence and parasitaemia-geometric mean parasite density (GMPD) relative to gravidity, gestational ages and age groups of participants, respectively. Prevalence was highest in the primigravida group while GMPD was highest amongst the secundigravida (Fig. 2). Prevalence was highest amongst the third-trimester pregnancies, while GMPD was highest in the second trimester (Fig. 3). Prevalence was at an equally high rate of 25% in the age groups ≤ 20 years, 21–25 years and 26–30 years with the highest GMPD recording in age group 21–25 years.

### Anaemia prevalence and risk factors

The prevalence of anaemia among pregnant women in the study was 81.4%. The mean (± sd) as seen in Table 2, PCV of parasitized pregnant women (30.01 ± 4.78) was significantly lower (χ^2^ = 65.34; P-value = 0.003) than that of non-parasitized pregnant women (30.9 ± 4.81). Further, pregnant women with malaria parasitaemia were 3.1 times more likely to present with moderate-to-severe anaemia compared to non-parasitized pregnant women (χ^2^ = 2.2; P-value = 0.13; OR: 3.1; 95% CI 0.85–0.11.36: RR:1.29; CI 1.03–1.60). Pregnant women with only primary education were about 6 times more likely to have moderate to severe anaemia compared to those with higher educational standards (χ^2^ = 2.2; P-value = 0.14; OR: 5.65; 95% CI 0.70, 45.33; RR:1.36; CI 1.10–1.67). Those in the first trimester of pregnancy were 1.19 times likely to present with moderate-to-severe anaemia than those in other trimesters 1.19 (χ^2^ = 0.09; P-value = 0.76; OR: 1.19; 95% CI 0.39–3.6; RR:1.05; CI 0.78–1.42). Pregnant women in the age group of 26–30 years were 2.7 times more likely to have moderate to severe anaemia compared to other age groups (χ^2^ = 4.02; P-value = 0.04; OR: 2.70; 95% CI 1.0–7.29; RR:1.28; CI 1.03–1.60). Interestingly, those who consumed medicinal herbs in pregnancy were approximately 1.8 times more likely to present with moderate to severe anaemia than women who did not consume medicinal herbs (χ^2^ = 1.44, P = 0.23, OR: 1.79; 95% CI 0.69–4.66; RR:1.17, 95% CI 0.92–1.47). However, participants in their first pregnancies, those who slept under LLIN the night before, those that had used anti-malaria in pregnancy and those that participated in IPT, were all less likely to have moderate to severe anaemia compared to those who had had more than one pregnancy, those that did not sleep under LLIN, those that had used anti-malaria and participated in IPT, respectively (Table 3).

**Table 3:** Behavioural factors associated with anaemia during malaria in pregnancy using univariate

Variable	Freq (%)	Anaemia		Mean (±sd) PCV	χ²	P-value	OR	95% CI	t-test	P-value
		None/Mild n (%)	Moderate/Severe n (%)							
Parasitaemia	22 (19.5)	3 (14.3)	19 (90.5)	26.8 (6.6)	2.33	0.13	3.11	0.85–11.36	−2.60	0.007
No parasitaemia	91 (80.5)	30 (32.6)	61 (66.3)	30.8 (6.0)						
Education										
None	5 (4.4)	1 (20.0)	4 (80.0)	29.6 (5.5)	0.00	1.00	1.68	0.18–15.66	–	–
Primary	13 (11.5)	1 (7.0)	12 (92.3)	28.9 (4.1)	2.22	0.14	5.65	0.70–45.33	0.26	0.40
Secondary	51 (45.1)	18 (35.3)	33 (64.7)	29.9 (6.2)	1.67	0.20	0.58	0.26–1.32	−0.12	0.46
Tertiary	44 (39.0)	13 (29.6)	31 (70.5)	30.6 (7.0)	0.004	0.95	0.97	0.42–2.23	−0.37	0.36
Pregnancy										
1st	32 (28.3)	11 (34.4)	21 (65.7)	29.6 (6.3)	0.58	0.45	0.71	0.30–1.71	−0.46	0.33
> 1st	81 (71.7)	22 (27.1)	59 (72.8)	30.2 (6.3)						
Trimester										
First	19 (16.8)	5 (15.1)	14 (17.5)	30.9 (7.2)	0.09	0.76	1.19	0.39, 3.61	–	–
Second	54 (47.8)	15 (45.5)	39 (48.7)	29.5 (6.3)	0.10	0.75	1.14	0.51, 2.57	0.75	0.23
Third	40 (35.4)	13 (39.4)	27 (33.8)	30.4 (5.9)	0.33	0.57	0.78	0.34, 1.81	0.26	0.60
Age (years)										
≤ 20	4 (3.5)	1 (25.0)	3 (75.0)	30.3 (5.4)	0.00	1.00	1.25	0.12–12.44	–	–
21–25	24 (21.2)	10 (41.7)	14 (58.4)	30.3 (7.4)	2.29	0.13	0.49	0.19–1.25	0.0	1.00
26–30	36 (31.9)	6 (16.7)	30 (83.3)	29.1 (5.1)	4.02	0.04	2.70	1.00–7.29	0.42	0.35
31–35	31 (27.4)	8 (25.8)	23 (74.2)	29.9 (7.1)	0.24	0.62	1.26	0.50–3.20	0.13	0.45
> 35	18 (159)	8 (44.5)	10 (55.6)	31.9 (5.6)	2.40	0.12	0.45	0.16–1.26	−0.53	0.31
ITN										
Use	15 (13.3)	5 (33.3)	10 (66.7)	30.5 (6.5)	0.14	0.71	0.80	0.25–2.55	0.28	0.39
Non-use	98 (86.7)	28 (28.6)	70 (71.4)	30.0 (6.3)						
Anti-malarial										
Use	55 (48.7)	17 (30.9)	38 (69.1)	29.4 (6.2)	0.15	0.70	0.85	0.38–1.92	−1.01	0.16
Non-use	58 (51.3)	16 (27.6)	42 (72.4)	30.6 (6.4)						
IPTp										
Use	32 (28.3)	12 (37.5)	20 (62.5)	30.3 (6.7)	1.49	0.22	0.58	0.24–1.39	0.29	0.39
Non-use	81 (71.7)	21 (25.9)	60 (74.1)	29.9 (6.1)						
Herb										
Use	33 (29.2)	7 (21.2)	26 (78.8)	28.2 (5.2)	1.44	0.23	1.79	0.69–4.66	−2.23	0.01
Non-use	80 (70.8)	26 (32.5)	54 (67.5)	30.8 (6.6)						

## Discussion

Earlier reports on the prevalence of anaemia in malaria and diagnostic performance of malaria detection methods compared with microscopy are auxiliary to this study [19, 20]. This study determined the association of prevention methods, socio-economic factors and some clinical criteria in pregnancy with the presence of malaria parasite and moderate to severe anaemia amongst the participants in this report.

Investigation revealed a low prevalence of malaria infection but a high prevalence of anaemia among pregnant women attending ANC in Lagos, South West Nigeria. The coverage of LLINs and IPT use during pregnancy was low. IPT use and education were well related to reduced prevalence of malaria and moderate to severe anaemia; mothers pregnant for the first time, those that had used anti-malarials or herbs during pregnancy were more likely to have falciparum parasitaemia.

In a similar study in Lagos, young maternal age (< 20 years) and primigravida had the same trend of association of increased risk of malaria infection as in this study. Maternal age (< 20 years) was however significantly associated with increased risk of malaria infection in that study, while primigravida was significantly associated with the same in this study. The present study similarly agreed with LLIN use not being associated with a reduction in malaria infection among study participants. Contrarily, however, low educational level was not associated with infection in that study as it is in this study [21].

In another study from the north of Nigeria, a similar association of higher odds was found with primigravida and a lack of education but unlike this current study, non-usage of LLINs was listed as increasing the odds of being infected with malaria [22]. LLINs are associated with reduced risk of malaria infection in several studies [22–25], although, others aligned with non-association or even significantly higher presence of parasitaemia with use, as observed in this study [26–28]. It has been suggested that the contrary to the expected association of higher prevalence with LLIN use observed in this study may be due to low ownership of LLINs in the communities lived in by participants. The community coverage of LLINs in the study may have been inadequate to confer a collective protective effect. LLIN use in the present study population study was low at 15% and the study was at a time when the national utilization of LLINs was 10% [15]. Furthermore, evidence suggests that ‘possession’ and ‘sleeping under LLIN the night before’ may not be satisfactory indicators to register protection by LLINs. Indicators of physical integrity and bio-efficacy, including good use, good physical integrity and biological efficacy of LLINs, have been shown to contribute to LLIN efficacy against malaria infection in the first trimester of the pregnant women studied [29]. Future investigations should seek to capture information on the age of LLINs, the number and size of holes in them (proportionate hole index (PHI)), their appropriate use, as factors in the measure of LLIN efficacy [30].

Education is an important predictor of how positive health status of individuals and extent of education in a population confers advantages in public health [31]. In this current study, the odds of having malaria infection was less amongst the educated compared to non-educated. In the case of anaemia, the odds of having moderate to severe anaemia amongst pregnant women with only a primary school education was 5.6 times compared to women of other educational qualification. That education confers knowledge is evidenced in a reported knowledge attitude and practice (KAP) study attributing knowledge of cause and symptoms of malaria to reduced infection rates among educated respondents compared to non-educated counterparts [23]. Other studies that have shown a positive association of a higher level of education with reduced odds of infection with malaria parasite abound in the literature [7, 32]. Anti-malarial use was 2.3 times more significantly likely to have malaria parasite than non-use in this study, as witnessed in an urban Colombian study where individuals receiving anti-malarial treatment in the previous month had around twice the risk of being infected as compared with those without treatment [33] A similar phenomenon was recorded in a Ghanaian community where the odds of malaria infection among those who received anti-malarial drugs 1 week before was 4.03 times more than in those who did not receive anti-malarials [34]. Over-prescription, over-diagnosis and self-medication are concurrent challenges known to contribute to chronic malaria infection [35, 36].

Anaemia was highly prevalent amongst the pregnant women surveyed in this study as reported [20]. Women who had malaria infection, those with only primary education and women who used herbs during pregnancy, had a higher odds of moderate to severe anaemia compared to other pregnant women in this study. On the other hand, the odds for women that used LLIN the night before and those that participated in IPT in pregnancy; to have moderate to severe anaemia were less than those that did not. The high burden of severe anaemia in pregnancy seen in this study is a well-established association with non-use of LLIN and IPTp in endemic regions [5, 37, 38]. Urban residence, some level of education and upper wealth quantile had reduced odds of moderate to severe anaemia in a Tanzanian study of women of reproductive age and showed some similarity with this study [39]. A traditional home birth study in Benin City, Nigeria, observed that malaria and the use of herbal remedies individually increased the odds of anaemia in pregnancy comparable to this study [40] and a Burkina Faso study of asymptomatic malaria cases that correlated with anaemia in pregnant women corroborated the same observation. The prevalence of anaemia at 71% was lower than in this study compared to the Benin study, perhaps because participants were asymptomatic compared to symptomatic participants recruited in the present study [41]. Malaria infection not only causes clearance of opsonized, intact, infected RBCs and ruptured cells after completion of the parasite’s intra-erythrocytic life cycle, but also clearance of uninfected cells. It is estimated that 10 uninfected cells are cleared from circulation for every infected cell, making it a crucial mechanism for the development of malarial anemia [42]. LLIN use the night before did not confer reduced risk to anaemia in observations from the Tanzanian demographic health survey contrary to observations in this study where protection was conferred against moderate to severe anaemia. That LLIN use conferred protection from moderate to severe anaemia but not from parasite infection herein may imply that LLINs nevertheless played a role in preventing malaria infection that has gone undetectable and which would have yet resulted in anaemia in this study [39]. IPT use in pregnancy has been associated with improved anaemia status in this and several other studies in Uganda [43], Ghana [44–46] and other countries [47], while administration of IPT was inconsequential in the development of maternal anaemia in other studies [48]. Herbal remedy users were 33% in this study similar to one-third rate observed in an Ibadan study of determinants of self-medication practices among pregnant women [49], but less than 67.5% in a study covering three geopolitical regions in Nigeria [50] and 68% seen in Abeokuta [51]. Association of herbal remedy use as a risk factor of moderate to severe anaemia is not well covered in literature and offers the opportunity to answer a cause/effect question considering the high prevalence of both the practice and condition in Nigeria. It is rational to infer that sub-therapeutic anti-malarial activities of herb teas results in chronic malaria which would likely result in anaemia. As was observed in the association of status of education with malaria infection, poor or no education is seen to increase the odds of moderate to severe anaemia in this study. Similar results associating education of women with less odds of having moderate to severe anaemia was also reported by other studies in Tanzania [39, 52, 53].

## Conclusion

Increased awareness of cause, symptoms and prevention of malaria and moderate to severe anaemia is necessary to protect pregnant mothers in the populace. Investigative research should be conducted to enquire into the proper utilization and physical integrity of LLINs in circulation amongst pregnant women, while intervention that will ensure rational anti-malarial drug use and discourage herbal medication in pregnancy should be implemented.

## Data Availability

The datasets used and/or analysed during the current study are available from the corresponding author on reasonable request.

## Abbreviations

OR: Odds ratio
CI: Confidence interval
IPTp: Intermittent preventive treatment in pregnancy
SD: Standard deviation
SP: Sulfadoxine-pyrimethamine
DOT: Directly observed therapy
ANC: Antenatal care
LLINs: Long-lasting insecticide nets
RBC: Red blood cells
LBW: Low birth weight
IPT: Intermittent preventive treatment
SP: Sulfadoxine-pyrimethamine
HPF: High powered field
PCV: Packed cell volume
EDTA: Ethylenediaminetetraacetic acid
FMP: Falciparum malaria parasite
GMPD: Geometric mean parasite density
ACT: Artemisinin-based combination therapy
